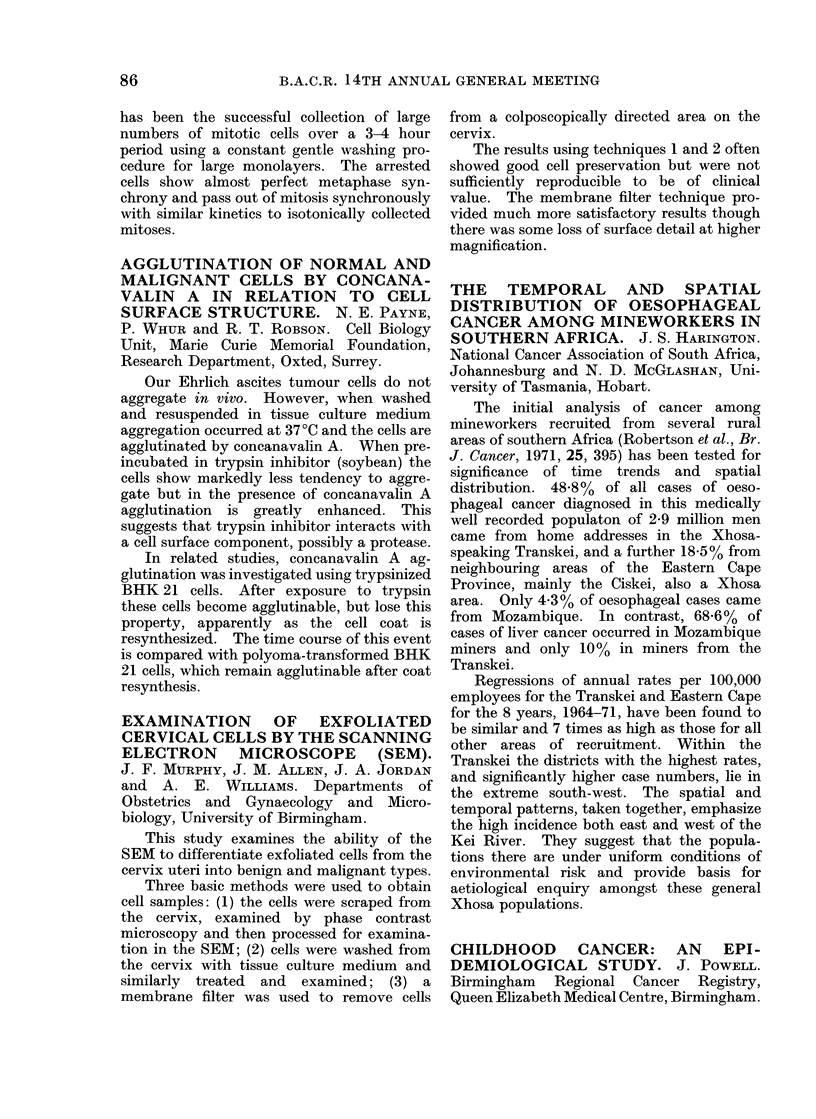# Agglutination of normal and malignant cells by concanavalin A in relation to cell surface structure.

**DOI:** 10.1038/bjc.1973.107

**Published:** 1973-07

**Authors:** N. E. Payne, P. Whur, R. T. Robson


**AGGLUTINATION OF NORMAL AND MALIGNANT CELLS BY CONCANAVALIN A IN RELATION TO CELL SURFACE STRUCTURE.** N. E. PAYNE, P. WHUR and R. T. ROBSON. Cell Biology Unit, Marie Curie Memorial Foundation, Research Department, Oxted, Surrey.

Our Ehrlich ascites tumour cells do not aggregate *in vivo*. However, when washed and resuspended in tissue culture medium aggregation occurred at 37 °C and the cells are agglutinated by concanavalin A. When preincubated in trypsin inhibitor (soybean) the cells show markedly less tendency to aggregate but in the presence of concanavalin A agglutination is greatly enhanced. This suggests that trypsin inhibitor interacts with a cell surface component, possibly a protease.

In related studies, concanavalin A agglutination was investigated using trypsinized BHK 21 cells. After exposure to trypsin these cells become agglutinable, but lose this property, apparently as the cell coat is resynthesized. The time course of this event is compared with polyoma-transformed BHK 21 cells, which remain agglutinable after coat resynthesis.